# Interaction of *Staphylococcus aureus* and *Candida albicans* with surface-modified silica studied by ultra-high frequency acoustic wave biosensor

**DOI:** 10.1039/d4ra05532b

**Published:** 2024-09-18

**Authors:** Brian De La Franier, Michael Thompson

**Affiliations:** a Department of Chemistry, University of Toronto 80 St. George Street Toronto ON M5S 3H6 Canada m.thompson@utoronto.ca

## Abstract

In this work the bacteria *S. aureus* and fungi *C. albicans* were allowed to interact with quartz-based biosensor devices under different flow rates, with and without an anti-fouling coating. These experiments were conducted in order to determine if the level of fouling observed was affected by the flow rate. The biosensor used was an ultra-high frequency acoustic wave device (EMPAS) for investigation of device surface initial interaction of *S. aureus* or *C. albicans* under flow of PBS buffer at flow rates between 50 and 200 μL min^−1^. Surface-bound microbes were also visualized by fluorescence microscopy following these experiments. *S. aureus* bacteria was able to foul the bare quartz sensors at each flow rate tested, with the greatest degree of fouling observed at a flow rate of 100 μL min^−1^. *C. albicans* showed far less fouling of bare devices with the maximum fouling observed at a flow rate of 75 μL min^−1^. Antifouling MEG-OH coated sensors showed greatly reduced fouling for *S. aureus*, with between a 90 and 99% reduction in observed frequency change depending on the flow rate used, and between 22 and 90% for *C. albicans*. Fluorescence images of the microbes following the experiments correlated well with the frequency data, showing a marked decrease in the amount of bacteria seen on MEG–OH–coated surfaces compared to controls.

## Abstract 2

In this work the bacteria *S. aureus* and fungi *C. albicans* were allowed to interact with quartz-based biosensor devices under different flow rates. The anti-fouling MEG-OH coating was able to reduce the fouling observed from the microorganisms.

## Graphical abstract

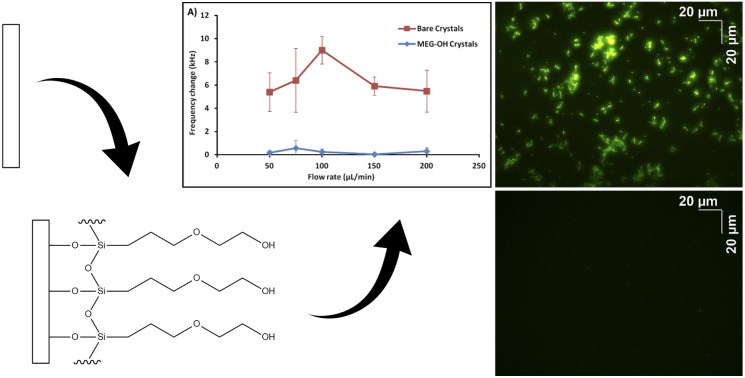

## Introduction

Microbial adhesion to surfaces and their subsequent biofilm formation can lead to a variety of technical issues which include, for example, the instigation of spreading disease and infection. This is especially problematic in the medical environment, where bacteria and other microbes can spread to patients *via* medical devices such as urinary catheters. These infections not only can result in medical complications and even death, but also result in high economic cost with regard to treatment.^1–4^ The ability of microbes to adhere to surfaces is affected by a wide variety of factors including surface charge, topology, free energy, hydrophobicity, and hydrodynamic flow.^5^ As such understanding how these factors affect microbial adhesion to surfaces is vital to not only understanding the mechanism of fouling of surfaces by microbes, but also to the development of strategies to reduce this effect.

Many strategies have been employed to reduce surface fouling including the imposition of various surface coatings such as polyethylene glycol, zwitterionic coatings, peptides, and polymer brushes.^6^ Surface topology and patterning has also been explored with respect to reducing microbial adhesion, though these efforts can be difficult to implement on real world devices as they can alter the function of the device.^7^ This is especially the case for sensor-based detection in bioanalytical chemistry, where changing the topology of the device surfaces can result in a complete breakdown with respect to function. As such ultra-thin and flat surface coatings are desired such that the anti-fouling coating has a minimal effect on the operation of the analytical sensor.

**Fig. 1:**
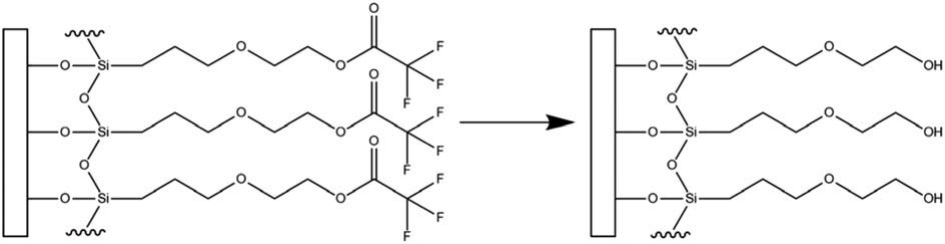
Transformation of surface bound MEG-TFA to MEG-OH. Adapted from “Detection of *E. coli* bacteria in milk by an acoustic wave aptasensor with an anti-fouling coating,” by Spagnolo S., De La Franier B., Davoudian K., Hianik T., Thompson M., *Sensors* (Basel), 2022, **22**, 1853 (https://www.mdpi.com/1424-8220/22/5/1853).

One such surface coating is hydroxyl terminated monoethylene glycol [2-(3-silylpropyloxy)-hydroxy-ethyl, (MEG-OH)], which is covalently bound to hydroxylated surfaces as a trifluoroacetate protected version [2-(3-trichlorosilylpropyloxy)-ethyl trifluoroacetate (MEG-TFA)] (Fig. 1).^8^ This coating has been studied on a variety of surfaces including polymers to prevent blood clotting^9–11^ and microbial adhesion,^12,13^ and stainless steel to prevent thrombogenesis.^14^ It has also been applied to high frequency acoustic wave biosensors, which use quartz-based devices as the sensing platform.

**Fig. 2:**
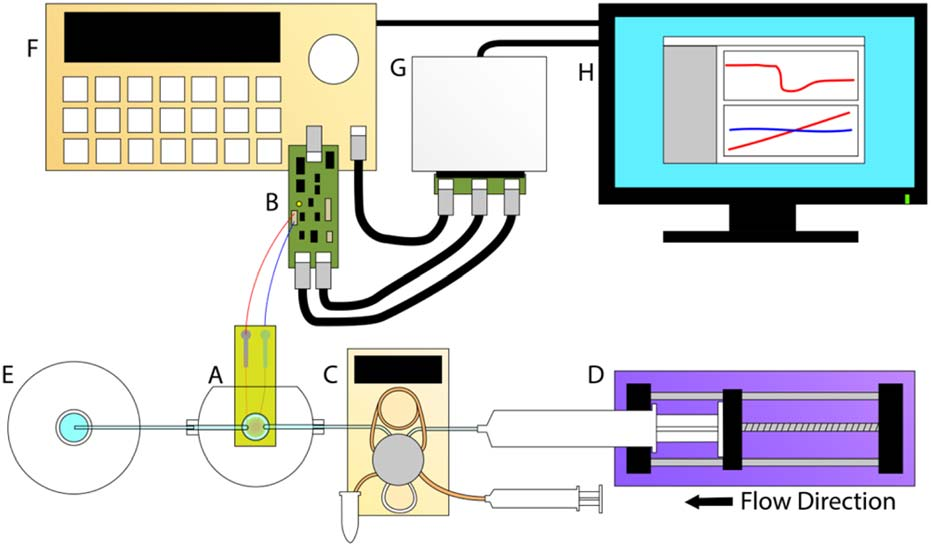
Schematic representation of the EMPAS biosensor system with (A) flow-through cell with driving coil and crystal, (B) control board, (C) Eppendorf auto-injector with loop, (D) syringe pump, (E) waste bottle, (F) signal generator, (G) National Instruments MyDAQ, and (H) computer interface.

The high frequency sensor used in this work, the electromagnetic piezoelectric acoustic sensor (EMPAS), is an acoustic wave biosensor that belongs to a class of quartz crystal microbalances (QCM) and surface acoustic wave (SAW) sensors. First developed in the early 2000s, the EMPAS system is capable of operation at high frequencies in the several hundred MHz, where traditional QCM devices typically operate in the tens of MHz.^15^ Multiple studies have been performed using the EMPAS to study anti-fouling coatings, typically against serum in biosensing investigations and typical flow rate conditions of 50 μL min^−1^.^16–19^ The system was also recently modernized to allow for use with modern operating systems, and make it easier to operate (Fig. 2).^17^

Previously the EMPAS system was used to investigate the ability of *Pseudomonas aeruginosa* PAO1 to foul both bare and MEG-OH modified quartz under different flow conditions.^20^ This work found that typically higher flow rates reduced the amount of fouling observed by the bacteria, with the exception of a flow rate of 75 μL min^−1^, which was found to exhibit higher levels of fouling than the slower 50 μL min^−1^ flow rate. This work also found that MEG-OH coated crystals showed significantly reduced fouling by *Pseudomonas* with between an 85 and 97% reduction in fouling observed depending on the flow rate. This present work aims to significantly expand on this previous effort to study the effect of flow rate and MEG-OH surface coatings on the bacteria *Staphylococcus aureus*' and the fungus *Candida albicans*' ability to foul the EMPAS biosensor crystals.

## Experimental

### Materials

MEG-TFA was synthesized according to previously published methods.^8^ Anhydrous toluene, sodium dodecyl sulfate, sulfuric acid, 30% hydrogen peroxide, Na_2_HPO_4_, TWEEN-20, glutaraldehyde, and NaCl were purchased from Sigma-Aldrich (St. Louis, MO, USA). Ethanol and acetone were obtained from Caledon Laboratory Chemicals (Georgetown, ON, Canada). Sytox Green and LB were purchased from U of T Medstore (Toronto, ON, Canada). All chemicals were used without further purification. Adhesion experiments were carried out with *Staphylococcus aureus* KR3 and *Candida albicans* SC5314. Quartz crystals (AT-cut, 13.5 mm in diameter, 20 MHz fundamental frequency) were purchased from Lap-Tech Inc., Bowmanville, Ontario.

### Cleaning and modification of quartz crystals

EMPAS quartz crystals were individually placed in test tubes and rinsed with 1% sodium dodecyl sulfate (SDS) aqueous solution, followed by sonication in 1% SDS solution (5 min). The crystals were then rinsed again with 1% SDS. This process was repeated with 95% ethanol, then with acetone, followed by a final rinse with distilled water.

The tubes containing the crystals were then submerged in a water bath held at 95 °C, and piranha solution was added (3 : 1 sulfuric acid : 30% hydrogen peroxide). After 30 minutes the piranha solution was removed and the crystals were rinsed thoroughly with distilled water. They were then dried under a gentle stream of nitrogen, and placed in an oven held at 180 °C for 45 minutes to finish drying. The crystals were then subjected to plasma cleaning under low pressure atmosphere for 5 minutes. Bare crystals were then stored for future use, while crystals to be modified were placed in a humidity chamber held at 70% humidity to hydrate the surface overnight.

The crystals for modification were transferred pre-silanized test tubes (overnight solution of 1% trichloro(octadecyl)silane in toluene), and brought into a glovebox under inert nitrogen atmosphere. A solution of MEG-TFA was prepared (1 : 1000 MEG-TFA : anhydrous toluene) and added to each crystal (2 mL per crystal). The tubes were sealed and removed from the glovebox and placed on a rotator at 120 rpm for 90 minutes. Following modification the crystals were cleaned by rinsing them with toluene, followed by sonication in toluene for 5 minutes. They were then rinsed with 95% ethanol, before being submerged in a 50% ethanol solution and placed on a rotator overnight (120 rpm) for deprotection. The deprotected crystals were then rinsed with 95% ethanol, followed by acetone, and dried under nitrogen to yield MEG-OH coated crystals.

### EMPAS Experiments

EMPAS experiments were carried out using similar methods to those previously described.^11^ A bare or MEG-OH modified crystal was placed in the custom flow-through cell, and secured using a screw clamp, creating a sealed chamber with an 83 μL volume through which solutions can flow over the crystal. A solution of PBS (10 mM Na_2_HPO_4_, 154 mM NaCl, pH 7.4) was pushed through an Eppendorf auto-injector, into the flow-through cell, and then into a waste receptacle using a syringe pump. In order to observe differences in fouling due to flow rate the flow rate was set to a value between 50 and 200 μL min^−1^. Once under flow the signal generator and control board are able to drive the coil, which in turn causes the quartz crystal to resonate at its fundamental frequency. Measurements were performed at the crystals 43rd harmonic (∼864 MHz), using custom computer software and a National Instruments MyDAQ control board. Once a stable baseline was observed the 250 μL injection loop was filled with *S. aureus* or C. albicans (overnight culture at 37 °C in Lysogeny broth (LB)), or Lysogeny broth for control, and immediately injected into the system. PBS flow was continued for 30 minutes following injection.

Cleaning was done by using a syringe to pass 12% bleach (1.5 mL), followed by 70% isopropanol (1.5 mL), then distilled water (1.5 mL) through the filling tubes. Air was then pushed through the filling tubing to remove any remaining water.

### Fluorescence imaging

Crystals were removed from the flow chamber and 1% glutaraldehyde in 0.9% NaCl solution was pipetted onto the surface to fix any microbes to the crystal (100 μL). After 30 minutes this was removed and 0.05% TWEEN 20 solution was pipetted onto the crystal (100 μL). After 10 minutes this solution was removed and Sytox Green in PBS buffer was added to the surface (50 μL). After 30 minutes the crystals were rinsed with 0.9% NaCl solution, and gently dried with nitrogen. During this time the EMPAS main tubing and flow chamber were cleaned using the same process as described in the previous section.

After staining, the crystals were imaged using an OMAX Epi-fluorescence microscope using a 40× objective lens under a green filter. Pictures of the crystal surface were taken at random locations across the surface to get a comprehensive idea of the total surface fouling.

## Results

**Fig. 3:**
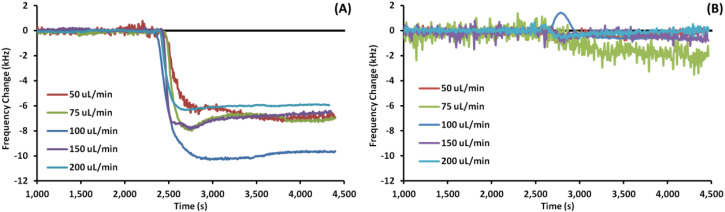
Example EMPAS runs for (A) bare quartz crystals and (B) MEG-OH coated quartz crystals at various flow rates with injection of 250 μL *S. aureus* in LB at ∼2500 seconds. Data normalized to baseline frequency.

Fig. 3 shows example EMPAS measurements of bare (A) and MEG-OH (B) coated crystals during injection of *S. aureus* in LB at each flow rate. For each flow rate the run that showed a frequency change closest to the average of all experiments at that flow rate was chosen as the example. The data was normalized to the baseline frequency, and lined up to an injection time of ∼2500 seconds. As can be seen by the example runs the frequency remains quite stable after bacterial injection during the PBS wash-off, regardless of the flow rate.

**Fig. 4:**
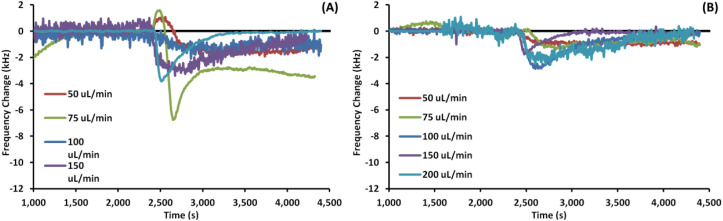
Example EMPAS runs for (A) bare quartz crystals and (B) MEG-OH coated quartz crystals at various flow rates with injection of 250 μL *C. albicans* at ∼2500 seconds. Data normalized to baseline frequency.

Fig. 4 shows example EMPAS measurements of *C. albicans* against (A) bare crystals and (B) MEG-OH modified crystals. As with the previous case, the measurement that showed the closest change to the average was chosen for each flow rate, and the data was normalized to baseline with the injection set to ∼2500 seconds. Unlike the experiments with *S. aureus* far more wash off is observed for *C. albicans* at most flow rates.

Control measurements of LB without any microbes were previously published.^20^ LB over bare crystals resulted in changes of 1.26, 1.28, 1.85, 0.69, and 0.45 kHz at flow rate of 50, 75, 100, 150, and 200 μL min^−1^ respectively, while no fouling was observed for LB over MEG-OH coated crystals.

**Fig. 5:**
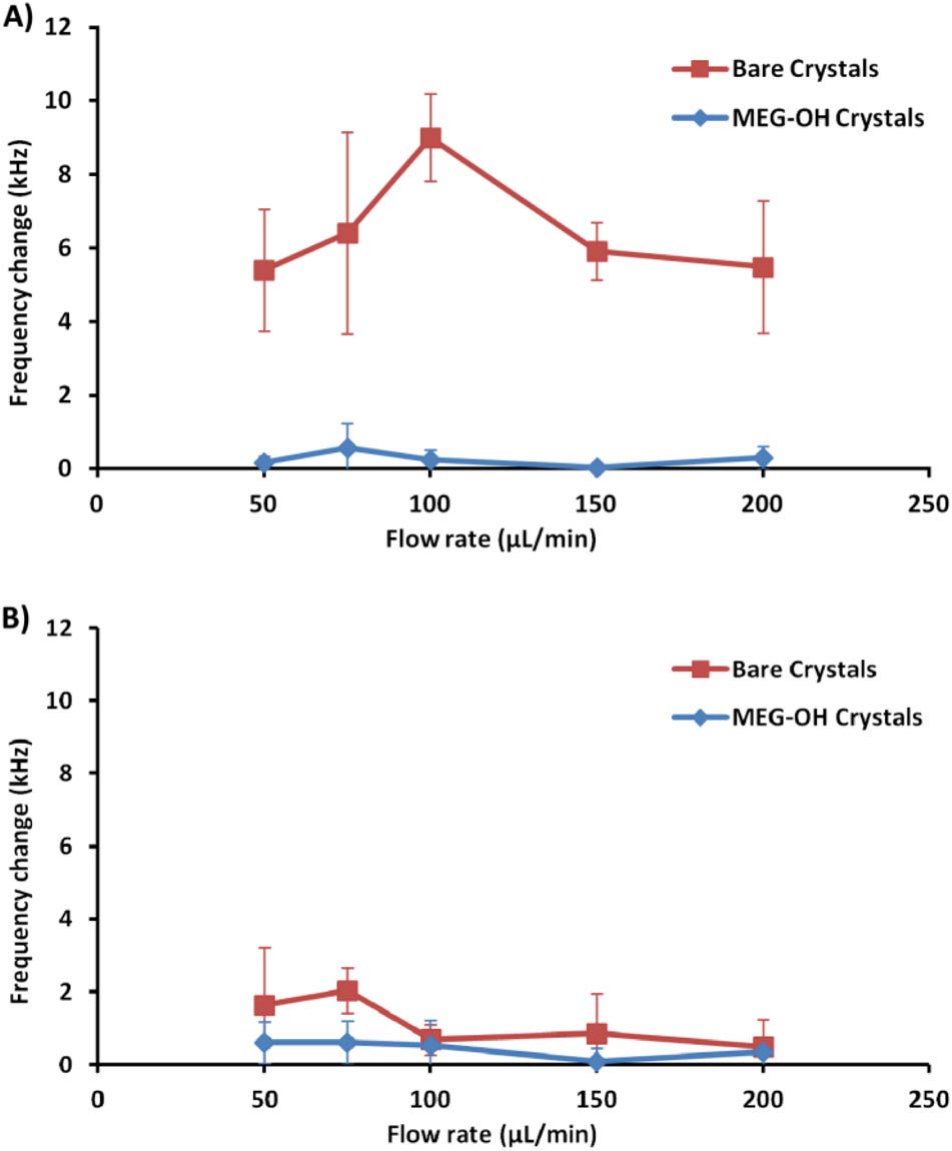
The data from Tables 1 (A) and 2 (B) visualized as line charts.

A minimum of 3 measurements were performed at each flow rate for bare and MEG-OH coated crystals against each of *S. aureus* and *C. albicans*. The frequency change was calculated as the average frequency immediately prior to injection minus the average frequency 30 minutes after injection. Table 1 contains the averages for these runs for *S. aureus*, as well as the percent reduction in fouling observed as a result of the MEG-OH coating. MEG-OH decreased the amount of fouling by *S. aureus* at every flow rate tested, by between 90 and 99%. Interestingly the greatest degree of fouling observed on bare crystals by *S. aureus* occurred at a flow rate of 100 μL min^−1^, while for MEG-OH coated crystals the greatest degree of fouling was observed at 75 μL min^−1^. The lowest degree of fouling for bare crystals was observed at 50 and 200 μL min^−1^, while the greatest reduction in fouling by MEG-OH was observed at a flow rate of 150 μL min^−1^. This data from Table 1 is visualized in Fig. 5A.

**Table 1:** Average frequency difference from baseline to 30 minutes after injection of 250 μL *S. aureus* over bare and MEG-OH coated crystals at various flow rates, and the percent reduction in frequency change caused by the MEG-OH coating

Flow rate (μL min^−1^)	Bare crystal Δfrequency (kHz)	MEG-OH crystal Δfrequency (kHz)	Reduction in change
50	5.40 ± 1.67	0.17 ± 0.17	97%
75	6.41 ± 2.75	0.57 ± 0.67	90%
100	9.01 ± 1.18	0.24 ± 0.28	96%
150	5.91 ± 0.78	0.04 ± 0.09	99%
200	5.49 ± 1.81	0.30 ± 0.30	95%

The average frequency changes for *C. albicans* can be found in Table 2. Unlike *S. aureus*, a very small frequency change was observed for *C. albicans* fouling of bare crystals. Oddly at higher flow rates the frequency change observed by *C. albicans* was lower than the change for LB control runs. Despite this lower level of observed fouling, MEG-OH was still able to reduce the amount of fouling observed. The greatest degree of fouling for bare crystals was observed at a flow rate of 75 μL min^−1^, with the least amount of fouling observed at 200 μL min^−1^. For MEG-OH modified crystals the greatest frequency change observed occurred at 75 μL min^−1^, while the lowest degree at 150 μL min^−1^. MEG-OH provided the greatest reduction in frequency change at 150 μL min^−1^, and performed most poorly at 100 μL min^−1^. The data from this table is visualized in Fig. 5B.

**Table 2:** Average frequency difference from baseline to 30 minutes after injection of 250 μL *C. albicans* over bare and MEG-OH coated crystals at various flow rates, and the percent reduction in frequency change caused by the MEG-OH coating

Flow rate (μL min^−1^)	Bare crystal Δfrequency (kHz)	MEG-OH crystal Δfrequency (kHz)	Reduction in change
50	1.62 ± 1.59	0.60 ± 0.57	63%
75	2.02 ± 0.62	0.60 ± 0.58	70%
100	0.68 ± 0.41	0.53 ± 0.67	22%
150	0.85 ± 1.09	0.08 ± 0.37	90%
200	0.49 ± 0.74	0.34 ± 0.37	30%

**Fig. 6:**
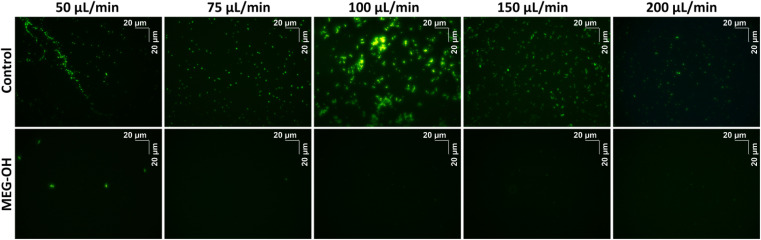
Florescent images of bare (top row) or MEG-OH coated (bottom row) EMPAS crystals following *S. aureus* exposure at various flow rates. Images were taken under green filter with a 40× objective lens.

**Fig. 7:**
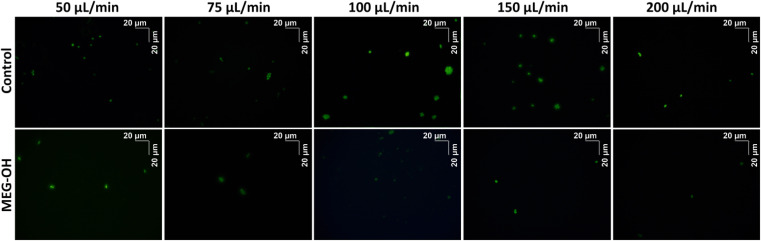
Florescent images of bare (top row) or MEG-OH coated (bottom row) EMPAS crystals following *C. albicans* exposure at various flow rates. Images were taken under green filter with a 40× objective lens.

Additionally, for each flow rate a bare and a MEG-OH coated crystal was removed from the crystal holder, stained with Sytox Green, and imaged using a fluorescent microscope to visualize the microbes. Each crystal was imaged 10 times at random locations, and an example representative image for each flow rate was chosen (Fig. 6 and 7). The average amount of *S. aureus* seen in the images correlates well with the frequency changes seen at each flow rate. For *C. albicans*, despite the measured frequency changes often being lower than those of the LB control runs, a fair number of cell clusters can be seen on the bare crystals. The number and size of cultures observed on MEG-OH crystals was typically lower in the presence of MEG-OH compared to bare crystals, agreeing with the frequency change reductions measured by the EMPAS system.

## Discussion

As with previous work studying *Pseudomonas aeruginosa* fouling of EMPAS crystals,^20^*S. aureus* was able to readily foul bare quartz crystals during the brief exposure time of these experiments. At each flow rate a frequency change of at least 5 kHz was observed following injection of *S. aureus* overnight culture onto the bare crystals. Oddly the amount of fouling observed increased from a flow rate of 50 μL min^−1^ to 100 μL min^−1^, and then decreased at higher flow rates. This could be due to the bacteria fouling the injection tubing at lower flow rates before reaching the crystal's surface, while higher flow rates allow more bacteria to reach the crystal surface. At flow rates above 100 μL min^−1^ the amount of exposure time of the bacteria to the crystal surface decreases, giving the bacteria less time to foul the surface. It is unlikely the shear stress caused by the fluid velocity at the surface has much of an effect on *S. aureus* surface fouling, since the frequency change remained stable following injection. If the shear stress from the fluid flow had an effect on the fouling, an increase in frequency should have been observed after the initial fouling due to bacteria being “washed” off the surface, but this is not the case.

For MEG-OH coated crystals a large decrease in *S. aureus* fouling was observed, with a frequency change reduction of between 90 and 99% compared to bare crystals depending on the flow rate. This agrees well not only with the previous EMPAS experiments with *P. aeruginosa*, but with previously published studies of the MEG-OH anti-fouling effect against bacteria on polyurethane.^12^ In that work MEG-OH was better able to reduce *S. aureus* fouling on polyurethane compared to *P. aeruginosa*. This holds true on quartz where *P. aeruginosa* fouling was reduced between 85 and 97%, while a similar reduction in fouling was observed against *S. aureus*.

Another factor to note is the control measurements where LB alone was injected over the EMPAS crystals.^20^ In these measurements the LB itself did foul the bare EMPAS crystals, resulting in frequency changes between 0.5 and 2 kHz against bare crystals depending on the flow rate, with no fouling observed against MEG-OH coated crystals. At each flow rate this was much lower than the frequency change observed when *S. aureus* was present in the injection, and account for less than 25% of the observed fouling at each flow rate. In the case of MEG-OH coated crystals no fouling was observed with only LB injected, with each signal returning to baseline after 30 minutes of flow regardless of flow rate. Even accounting for the small amount of fouling caused by LB on the bare crystals, MEG-OH coated crystals still show a large degree of anti-fouling against *S. aureus* bacteria.

Another area where this study compared favourably to the previous EMPAS study is the variation in signal change observed between runs. For bare EMPAS crystals the variation between runs was quite high, as much as 2.75 kHz at a flow rate of 75 μL min^−1^. This variation was much lower in the case of MEG-OH coated crystals, with a maximum variation of 0.67 kHz also at a flow rate of 75 μL min^−1^. As such not only does MEG-OH reduce the amount of fouling observed, but it allows for more consistent measurements with less variation, something very important for accurate and reproducible analytical measurements.

Unlike the previous work with *P aeruginosa*, this study also includes fluorescence imaging of surface bound *S. aureus* or *C. albicans* after EMPAS measurement. Each crystal had any surface bound microbes fixed to the surface following the 30 minutes PBS wash-off in the EMPAS, and was stained with Sytox Green for visualization by fluorescence microscopy. Ten images using a 40× objective lens were taken of each crystal at random location across the surface. For bare crystals *S. aureus* were seen consistently across the crystal surfaces at each flow rate, though some variation in the amount of bacteria was seen. The average amount of surface bacteria seen at each flow rate correlated well with the frequency changes measured using the EMPAS, with the most bacteria seen at a flow rate of 100 μL min^−1^, and the least at flow rates of 50 and 200 μL min^−1^. The bacteria was also observed to clump together more at 100 μL min^−1^, while more individual bacteria were seen at the other flow rates.

In the case of MEG-OH coated crystals very few bacteria were observed on each surface, with many of the recorded images showing zero or only a few bacteria within the field of view. Images where bacteria could be seen were chosen for this publication (Fig. 6). As with the EMPAS measurements, a marked decrease in bacteria was seen on the MEG-OH coated crystals compared to bare crystals at each flow rate. As well there was very little clumping of bacteria seen on MEG-OH coated surfaces, with most bacteria observed being alone where they were on the surface.

For *C. albicans* exposed to bare crystals clumps of cells could be seen across the crystal surface at flow rates of 100 and 150 μL min^−1^, with individual cells also being present. At flow rates of 50 and 75 μL min^−1^ there were fewer cell clumps observed, but smaller clumps and individual cells were still common across the surfaces. At the fastest flow rate of 200 μL min^−1^ only a small number of individual cells were observed in each image. Despite the large number of cell clumps visually observed on the crystal surfaces at the 100 and 150 μL min^−1^ flow rates, the frequency changes were quite low, lower than the control runs in the case of 100 μL min^−1^. Additionally the observed frequency change for lower frequencies was greater than these frequencies, even though far more cells could be seen on the surface visually at these two higher flow rates. This suggests that surface bound *C. albicans* clusters exhibit some effect on the slip of the crystal, though further work would need to be done to determine if that is the case.

For both the frequency data and the fluorescence images, a reduction in fouling was observed for MEG-OH coated crystals. The level of reduction in fouling was lower than that observed for *S. aureus*, with between 22 and 90% reduction seen depending on flow rate, compared to 90 to 99% for *S. aureus*. This is in agreement with previously published work, which demonstrated a greater ability of MEG-OH to reduce *S. aureus* fouling compared to *C. albicans*.^12^ This could also be due to *C. albicans* showing a much lower frequency change on the control crystals, so any variation in the fouling of MEG-OH coated crystals had a much greater effect on the observed reduction in fouling compared to *S. aureus*. The overall differences in fouling observed between *S. aureus* and *C. albicans* on bare crystals is possibly due to differences in the outer membrane proteins of *S. aureus*,^21^ and the cell wall of *C. albicans*.^22^ These 2 species use difference proteins for surface adhesion and biofilm formation, which could act differently in interactions with quartz, though further research would be needed to verify this.

## Conclusions

At each flow rate studied with the EMPAS sensor MEG-OH was able to reduce the fouling observed by *S. aureus* and *C. albicans* on the sensor's quartz crystals. The reduction in fouling for *S. aureus* was found to be greater than the reduction for *C. albicans*, though this is likely due to the limited fouling of bare crystals observed for *C. albicans*. This work agrees with the previous EMPAS publication, where a flow rate of 150 μL min^−1^ was determined to be the best flow rate for reducing fouling using MEG-OH coatings. Significant anti-fouling was still observed at a flow rate of 50 μL min^−1^, so experiments where a longer interaction time between analytes of interest and their surface bound probes are needed this lower flow rate can still be used with limited fouling when MEG-OH coatings are used. Further work studying microbial interactions with surfaces using the EMPAS sensor should focus on the interaction time of microbes and surfaces under static conditions to determine the rate of bacterial surface fouling on bare and modified surfaces.

## Data availability

Data is available on request to either of the authors: m.thompson@utoronto.ca, brian.delafranier@mail.utoronto.ca.

## Author contributions

BD: conceptualization, investigation, writing – original draft, writing – review & editing. MT: writing – original draft, writing – review & editing, validation, supervision. All authors read and approved the submitted version.

## Conflicts of interest

The authors declare that they have no conflicts of interest.
